# The Age-Related Association of Movement in Irish Adolescent Youth

**DOI:** 10.3390/sports5040077

**Published:** 2017-10-02

**Authors:** Diarmuid Lester, Bronagh McGrane, Sarahjane Belton, Michael J. Duncan, Fiona C. Chambers, Wesley O’ Brien

**Affiliations:** 1 School of Education, Sports Studies and Physical Education, University College Cork, 2 Lucan Place, Western Road, Cork, Ireland; f.chambers@ucc.ie (F.C.C.); wesley.obrien@ucc.ie (W.O.B.); 2 School of Arts Education and Movement, Dublin City University, Institute of Education, St. Patrick’s Campus, Dublin, Ireland; bronagh.mcgrane@dcu.ie; 3 School of Health and Human Performance, Dublin City University, Dublin, Ireland; sarahjane.belton@dcu.ie; 4 Research Centre for Applied Biological and Exercise Sciences, Coventry University, Priory Street, Coventry CV1 5FB, UK; michael.duncan@coventry.ac.uk

**Keywords:** age, fundamental movement skills, functional movement screen, adolescent

## Abstract

**(1) Background:** Research has shown that post-primary Irish youth are insufficiently active and fail to reach a level of proficiency across basic movement skills. The purpose of the current research was to gather cross-sectional baseline data on Irish adolescent youth, specifically the prevalence of movement skills and patterns, in order to generate an overall perspective of movement within the first three years (Junior Certificate level) of post-primary education; **(2) Methods: **Data were collected on adolescents (N = 181; mean age: 14.42 ± 0.98 years), attending two, mixed-gender schools. Data collection included 10 fundamental movement skills (FMS) and the seven tests within the Functional Movement Screen (FMS™). The data set was analysed using the Statistical Package for Social Sciences (SPSS) version 20.0 for Windows; **(3) Results:** Overall, levels of actual mastery within fundamental and functional movement were low. There were statistically significant age-related differences observed, with a progressive decline as age increased in both the object control (*p* = 0.002) FMS sub-domain, and the in-line lunge (*p* = 0.048) test of the FMS™; **(4) Conclusion:** In summary, we found emerging evidence that school year group is significantly associated with mastery of movement skills and patterns. Results from the current study suggest that developing a specifically tailored movement-oriented intervention would be a strategic step towards improving the low levels of adolescent fundamental and functional movement proficiency.

## 1. Introduction

Research has established that levels of physical activity (PA) participation decline significantly during adolescence [1,2]. The ability to perform a variety of fundamental movement skills (FMS) may serve as a protective factor against this trend however [3,4], with empirical evidence suggesting that proficiency in FMS is positively associated with PA participation [4,5,6,7]. Therefore, strategies to improve PA participation may need to consider ensuring that adolescents have competency in basic movement patterns [8,9,10,11], at both a fundamental and functional movement level [12,13,14].

FMS are considered the basic observable building blocks, or precursor patterns of the more specialised, complex movement skills required to successfully participate in organised and non-organised games, sports and recreational activities [15,16]. Examples exhibited during sport, exercise and PA include running, hopping, skipping (locomotor), throwing, catching, kicking (object control), balancing, twisting and dodging (stability) [17,18]. Previous evidence suggests that children have the developmental potential to master most FMS skills by six years of age [18].

Children and adolescents who have established a base of FMS may possess some of the tools to be physically active [19], and with that, can potentially benefit from a lifetime of health-enhancing PA [18]. Crucially, competency in a range of FMS increases the likelihood of children and adolescents participating in different physical activities throughout their lives [6,20,21]. Conversely, those who lack FMS are at an increased likelihood to experience the consequences of “public failure”, or ridicule from peers [22], and subsequently may avoid participation in organised sports. In turn this can then serve to decrease their development toward a physically active lifestyle [23]. From a public health perspective, low motor competence and FMS performances amongst adolescent youth may relate to the escalating prevalence of childhood obesity [24] and weight status [25]. Movement skills are one of the few modifiable risk factors for the prevention of poor health outcomes [26].

Functional movement is another indicator of actual movement proficiency, as it relates to the body’s use of multi-planar and multi-joint movements, specifically those activating the core musculature region [12]. Previous research has reported low levels of functional movement among children and adolescents [12,27]. If such suboptimal functional movement strategies persist, there is a suggestion that this may lead to orthopaedic abnormality (e.g., arthritis, low back pain, osteoporosis) in later life [28].

The Functional Movement Screen (FMS™) has predominantly been used in injury-related research for assessing functional mobility and postural stability [13,14]. The FMS™ instrument was conceptualised as a tool that assesses movement patterns and functional movement capacity [13,14]. It is therefore logical to suggest that children who show high levels of functional movement, may also show higher levels of FMS proficiency, as functional mobility and postural stability underpin performances in the basic observable patterns of running, hopping, jumping, and throwing [29]. This suggestion is based on the assumption that strength, movement, flexibility and stability are prerequisites for fundamental skill performance, which the FMS™ purports to examine [29]. Although the FMS and FMS™ share some commonality as movement assessments, it is not a given that an individual who has high levels of skill proficiency in FMS will also score highly in the FMS™. Where this is the case, it may be an indication that they are making significant compensations that will, at best, only temporarily allow for high-level skill-related performance [30]. Cook et al. [13,14] identifies functional movement as the base of a movement pyramid with FMS, representing more advanced movement patterns, sitting on top of this. Thus, understanding or considering both fundamental and functional movement as two elements in a continuum of movement competence may provide a more insightful understanding within the motor development domain, by reflecting more accurately the skills and movements inherent in a wider range of sports and games in which adolescents participate.

Age, and thereby previous practice and experience of FMS, is an important factor in the development of FMS [31]. For example, a study of four object control skills found improvements were characterized by early, rapid gains at ages 9 to 10, beyond which development occurred at a slower rate for catching, throwing, and kicking, although striking development continued at a steady rate to an age of 14 years [19]. Understanding the trends in FMS competence by sex and age provides practitioners with valuable information to implement instructional and intervention strategies, curriculum development, and policy changes [31]. FMS and the process of motor development as a whole is age-related but not always age-determined [10,18]. Chronological age, for example, is a poor indicator of maturity due to the individuality and extreme variability of the growth process, particularly during later childhood and early adolescence [18]. However, it can also be difficult to determine biological maturity particularly for practitioners in the field. Similarly, most children, once they gain body control, can pass the FMS™ with minimal difficulty [30]. During adolescence and puberty, asymmetrical growth occurs between the legs and the upper torso. The lower extremity almost always demonstrates stiffness in the hips and ankles, including tightness in the lateral hip musculature and hamstrings which creates an obvious awkwardness to adolescent movement [30]. While many adolescents do subsequently rebalance themselves after puberty, some do continue to display poor movement patterns through adulthood [30]. Ultimately, identifying individuals with a suboptimal movement foundation [32], as well as any weaknesses and asymmetries could play a key role in enabling lifelong habitual PA and movement [33].

Gender-based differences are also apparent within the FMS literature, as males appear to have higher movement skill proficiency within the object control subset, when compared to females [7,10,34]. Gender based FMS™ differences have also been identified within adolescent populations, although the research in this area is currently limited. Previous research [12] found that males were on average better within the in-line lunge, active straight leg raise, trunk stability push-up and the rotary stability tests than females. Most recent research in Ireland found that female adolescents outperformed males in their overall functional movement, specifically six of the seven movements within the FMS™, with the exception of the trunk stability push-up [27].

The purpose of the current research was to gather cross-sectional baseline data on Irish adolescent youth, specifically in order to generate an overall perspective of movement during the initial three years (Junior Certificate level) of post-primary education. This study presents findings on the prevalence of mastery (displaying correct performance on all components of a fundamental movement skill and functional movement pattern) [10] for males and females, differentiated by school year group (first year through to third year). It was hypothesized that adolescents’ movement skills and patterns would be positively associated with school year group.

## 2. Materials and Methods

### 2.1. Participants and Setting 

A convenience sample of cross-sectional data was collected on Irish adolescent youth as part of the baseline study protocol. Students were grouped based on year of enrolment in the school. Ethical approval was provided by the Social Research Ethics Committee (SREC) of University College Cork (March 2016). Prior to the commencement of this school-based study, the leading researcher visited the principal of each of the participating schools, where a full brief and outline of the data collection was provided. Subsequent to the granted approval from school principals, information sheets and consent forms were then distributed to the selected class groups. Informed parental consent and child assent were the requirements for eligible participation in this study. Each school and participant was informed that their participation in the study was entirely voluntary, and that they were free to withdraw from the study at any time. In terms of the research rigour associated with school-based measurements, it is important to note that the principal investigators for this study are qualified post-primary specialist physical education (PE) teachers, as recognized by the Teaching Council of Ireland.

Consenting post-primary participants enrolled in years one to three (12–16 years) from two mixed-gender, non-fee-paying schools were invited to partake. Both post-primary schools involved in the research study were from the same suburban area in County Cork, within the province of Munster, Ireland. Two hundred and twenty-seven participants from the two schools were invited to participate in this study, with consent from 219 participants provided (97% of total sample). 

### 2.2. Data Collection

Prior to data collection, all thirteen field staff underwent a rigorous and robust 8 h field researcher training workshop in the measurement protocol associated with FMS and the FMS™. This involved an objective, criteria-informed process to ensure field staff were consistent in the administration and implementation of the respective gross motor skill and movement task(s). Baseline data, specifically the objective measurements of FMS and the FMS™, were collected on participants in their class groups (maximum *n* = 30) during a typical PE class. 

### 2.3. Measures

**Fundamental Movement Skills.** The following 10 FMS were assessed: run, skip, horizontal jump and vertical jump (locomotor, maximum score of 34); two-handed strike, stationary dribble, catch, kick, overhand throw (object control, maximum score of 40) and balance (stability, maximum score of 10), which combines to give an overall maximum raw score of 84. Process-oriented assessments of FMS were used in preference to product-oriented assessments because they identify more accurately specific topographical aspects of the movement [35,36]. Each of the ten FMS were assessed in conjunction with the observable, behavioural components from three testing batteries with established reliability and construct validity [17,37,38], namely the Test of Gross Motor Development (TGMD) [38] (skip), TGMD-2 [35] (run, horizontal jump, two-handed strike, stationary dribble, catch, kick and overhand throw) and the Victoria Fundamental Motor Skills manual [17] (balance and vertical jump). These instruments were selected to give an objective measurement of gross motor skill proficiency across a range of skills, including those skills particularly relevant to the Irish sporting context and PE environment [10].

Prior to participant performance, one trained field staff member provided an accurate demonstration and instruction of the skill to be performed. Procedures outlined in the TGMD-2 examiner’s manual [35] were closely adhered to within the assessment of the ten FMS during the selected PE period. To ensure participant consistency within skill performance, no feedback, verbal or otherwise, from any of the trained field staff were given during the testing. Participants performed the skill on three occasions, including one familiarization practice, and two performance trials, as reported in previous Irish adolescent movement skill data collection protocol [10,11,39]. The number of performance criterion varied from three to six across the range of selected FMS; all participants were given a ‘1’ for correct execution of a criterion and a ‘0’ for a failure on a criterion. For each FMS, the two performance trials were added together to get the total for each skill score which equated to the total of 84 across the ten skills [10]. Video cameras (3× Canon type Legria FS21 cameras; Canon Inc., Tokyo, Japan and 2× Apple iPads, Apple Inc., California, United States) were used to record each participant’s performance, and execution of the required skill. The distance and camera angles were at all times consistent; specifically, to ensure that the complete body movement was captured [10,11]. The use of video-recording is an important consideration in data collection, as it permits greater scrutiny and therefore accuracy of measurement precision [40]. The behavioural components of each skill were assessed at a later date by the principal investigators.

**Functional Movement Screen.** The Functional Movement Screen (FMS™) [13,14,41] is a pre-participation evaluation tool that comprises a series of movements designed to assess multiple domains of function, and the quality of movement patterns [42,43]. All seven components of the FMS™ were assessed: deep squat, hurdle step, in-line lunge, shoulder mobility, active straight-leg raise, trunk stability, push-up and rotational stability [41]. The test administration procedures, instructions and scoring process associated with the standardized version of the test [13,14] were followed in order to ensure accuracy in scoring [12,44]. Normative values have been established for the FMS™ in adolescent school-aged children [12]. Trained field staff utilised the pre-determined verbal instructions during testing. During data collection, each participant was again video-recorded, and given three attempts to perform the movement.

The FMS™ has a scoring range from zero to three for each individual test, with three being the optimum score [45]. If the participant at any time during the testing demonstrated or acknowledged pain, or discomfort, anywhere in the body, he/she received a score of zero, and the area was noted. A score of one was given to a participant if they were unable to complete the movement. If the participant had to use a compensation to perform the movement, a score of two was allocated. A maximum score of three was allocated if the participant performed the movement correctly without any compensation. Bilateral scores for five (hurdle step; in-line lunge; shoulder mobility; active straight leg raise and rotary stability) of the seven functional movements were also recorded, as a means to compare possible imbalances between the right and left sides of the body for participants. The lowest score for either side of the body within each movement contributed to the final scoring protocol. For each of the seven screening items, the highest score from the three trials was recorded, and used to generate an overall composite FMS™ score, with a maximum value of 21, as part of established and recommended protocol [13,46,47]. On account of the video-recording set-up for data collection, it should be noted that the principal investigators scored the optimum trial stringently at a later date, with each component test scored on an ordinal scale, and total composite score then calculated.

### 2.4. Data Analysis

Once data collection was complete but prior to data scoring, inter- and intrarater reliability was established on 10% of the data set. That is, two rater’s double coded 10% of the data to determine intrarater reliability, and both rater’s coded the same 10% of data to determine interrater reliability [36]. The two principal investigators were required to reach a minimum of 95% interrater agreement for all ten FMS and seven FMS™. The FMS and FMS™ data sets were analysed using SPSS version 20.0 for Windows. Participants with any missing data as a result of incomplete camera angle footage or otherwise were subsequently omitted from the data set and analyses. Descriptive statistics and frequencies for FMS and FMS™ at the skill and composite score levels were calculated. Age-related differences in overall and individual FMS/FMS™ performances were analysed using one-way analysis of variance (ANOVA), or the Kruskal–Wallis test in the case of non-parametric data. Chi-square tests for independence identified if any movement-based differences by school year group existed while effect sizes, based on Cramer’s V, were classified as small = 0.07, medium = 0.17 or large = 0.29. Statistical significance was set at *p* < 0.05.

## 3. Results

Of the participants, 108 were male (59.7%) and 73 were female (40.3%); 79 adolescents were in year one (43.6%), 43 adolescents were in year two (23.8%) and 59 adolescents were in year three (32.6%). The mean age of the participants was 14.42 ± 0.98 years (age range: 12.31–16.41 years old). The associated age-related sample provides the opportunity to compare and contrast the mastery levels of adolescents across three year groups. The results will be presented below separately for FMS and the FMS™.

### 3.1. Fundamental Movement Skills

**Figure 1:**
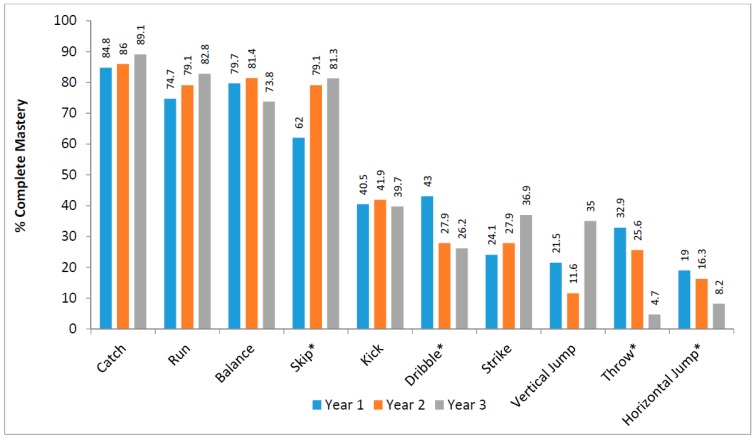
Percentage mastery of fundamental movement skills (FMS) by school year group. * *p* ≤ 0.05.

No participant, of those with full FMS data (n = 181), displayed a complete mastery level across all ten skills. The mean overall composite score was 68.72 (±7.54), out of a possible total of 84. The highest skill performance was the catch, with 86.6% of the total sample achieving complete mastery. The poorest performance was for the horizontal jump, where only 14.8% of all students achieved complete mastery. The percentage of students who displayed complete mastery in each of the ten FMS, differentiated by school year group, is shown in Figure 1, while the proportion of males and females in each school year group who completely mastered each behavioural skill component across all ten FMS is shown in Table 1.

Across school year groups, a Kruskal–Wallis Test revealed a statistically significant (*p* = 0.002) effect for FMS data, with a progressive decline observed in the object control subset from first through to third year. A series of chi-square tests for independence were also conducted to determine the associations between school year group and FMS. Significant associations in the horizontal jump (*p* = 0.040) and the skip (*p* = 0.003) were found with the former showing a progressive decline and the later a progressive improvement from first through to third year. A particularly large effect size (0.431) was found for the object control subset (*p* = 0.002), and the individual skills of the dribble (*p* = 0.012) and the throw (*p* = 0.001), with both skills showing a progressive decline from the first through to the third year.

### 3.2. Functional Movement Screen

**Figure 2:**
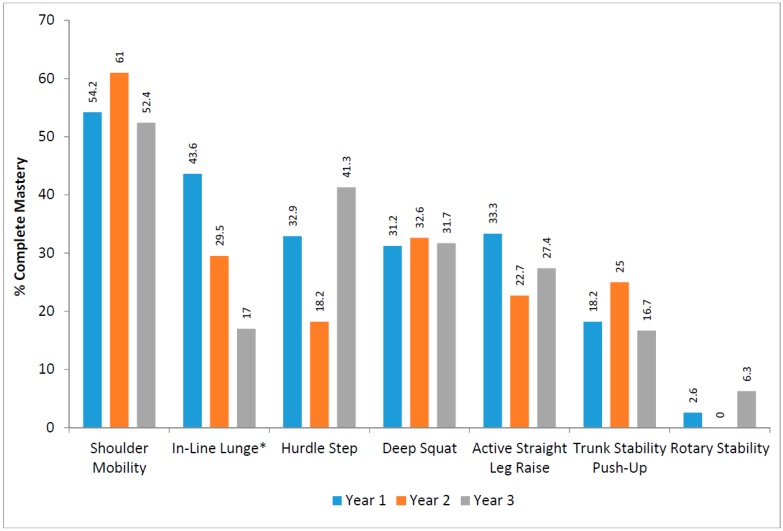
Percentage mastery of the Functional Movement Screen (FMS™) by school year group. * *p* ≤ 0.05.

No participant, of those with full FMS™ data (*n* = 152), achieved complete mastery across all seven tests (maximum score of three for all). The mean composite score was 14.05 ± 2.48 out of a possible total of 21. The percentage of students who displayed complete mastery in each of the seven screening measurements, differentiated by school year group, is shown in Figure 2, while the proportion of students in each school year group who completely mastered each component of all seven assessments of the FMS™ is shown in Table 2.

Across the school year groups, a one-way ANOVA found a statistically significant (*p* = 0.048) decline in the in-line lunge from first through to third year. A chi-square test for independence confirmed a significant association between the year group and the in-line lunge (*p* = 0.012), with a Cramer’s V (0.196) indicating a small to medium effect size.

## 4. Discussion

The aim of this study was to generate an overall perspective of movement during the initial three years (Junior Certificate level) of post-primary education. This will serve to heighten the reader’s understanding of the trends in movement proficiency. To the authors’ knowledge, this is the first study of its kind to combine both fundamental and functional movement assessment in an adolescent population. The cross-sectional baseline results highlight that a large proportion of adolescent youth are lacking both fundamental and functional movement skill proficiency. Specifically, no participant demonstrated overall mastery across the range of selected FMS and/or the FMS™, irrespective of the associated school-age year group breakdown. Irish adolescent youth may therefore be engaging in sport-specific skills, without learning the correct technique for the execution of basic skills and movement patterns [10]. This has potentially serious long-term consequences as it has been identified that keeping adolescents active has a greater impact on adult activity, as tracking improves with age [48].

Failure to develop proficient forms of fundamental movement has direct consequences for an individual’s ability to perform task-specific skills at the specialized movement phase [18]. The cohort assessed in this study ranged in age from 12 to 16 years, and therefore have the potential to be moving from the application to lifelong utilization stages of motor development within the aforementioned specialized movement phase [49]. However, overall skill execution is low amongst the selected cohort of adolescent youth. These findings support the most recent motor development literature within Ireland [8,10], suggesting that a ‘proficiency barrier’ [50] may exist, in which the acquisition of sport-specific skills may be hampered by not developing an initial base of mature skills during the fundamental movement phase of development [49]. The transition from one phase of development to another depends on the application of proficient patterns of movement to a wide variety of movement skills [18]. Many adolescents lag in their movement capabilities because of limited opportunities to regular practice, poor or absent instruction, and little or no encouragement, albeit a person may still be cognitively and affectively ready to advance to the specialized movement skill phase of development [18]. Interestingly, these overall low FMS findings are in line with most recent research carried out on adolescents in a different region of Ireland, which found that adolescents aged between 12 and 13 years entering their first year of post-primary physical education did not display proficiency across nine basic movement skills. [10].

The three poorest performed skills across school year group are the overarm throw, vertical and horizontal jump. Mastering proficient throwing and jumping fundamental movements requires considerable muscular strength and power [51]. It might seem plausible, therefore, as children move through adolescence that these particular skills would improve, in tandem with physiological muscular strength gains. The opposite is true, however, in this study with significant age-related declines observed in both throwing and jumping-related movements. Similar findings were observed in relation to poor levels of mastery in the vertical and horizontal jumps in a previous study amongst an adolescent cohort in Ireland [10], albeit in contrast to these findings, a study in Australia (using mastery and near mastery (MNM)) found that over 80% of adolescents, irrespective of gender, had reached MNM performance in the vertical jump [34]. A possible explanation for the findings in the present study could be that to develop muscular strength, children and adolescents need to be physically active and provided with the opportunities to engage in outdoor play [52]. It is well-established that the targeted adolescent population are becoming less physically active [53,54]. Another plausible explanation for this trend is that too much emphasis is being placed on competitive participation at an early age, potentially leading to a later demise of mature motor development during adolescence. Evidence would not suggest that competition should be abandoned, but that more opportunities be made available and greater emphasis placed on the development of efficient and effective movement [18,55].

Similar to the low levels of FMS proficiency observed in the present study, overall sports-related functional movement patterns were low amongst participants, which is consistent with other previously published functional movement adolescent literature [12,56,57]. Overall, the mean composite FMS™ raw score for this study was 14.05 (out of a possible 21), which is similar, albeit slightly lower than the mean values reported by Abraham et al., (2015) on 1005 mixed-gender adolescents in India. 

Interestingly, the in-line lunge was the only functional movement that showed a significant decline from first through to third year; the pattern that was evident for many of the FMS. This screening measurement assesses hip and ankle mobility and stability, quadriceps flexibility and knee stability [58]. Furthermore, the in-line lunge movement pattern is a component of deceleration and directional change, produced in exercise, activity and sport [45]. Although poor performance during this movement can be the result of several factors, further analysis at a behavioural component level identified limitations in the thoracic spine region. In fact, 67% of participants in their first year of post-primary school were able to keep the dowel in contact with their head, thoracic spine and sacrum on the right side of the body; this figure fell to 54.5% in second year, and 36.2% in third year. Similarly, on the left side of the body, the decline across school year groups was equally apparent. These findings are further magnified when we compare the observed low findings of the overarm throw across the varying school year groups. Evidence suggests that efficient throwing is very often a result of properly timed weight shifting from the back foot to the front foot [45]. This linear power transition turns into rotational power when the wave of energy generated in the lower body reaches the upper body, creating a throw [45]. Further evidence would also suggest that as adolescents progress through their schooling years, prolonged periods of sitting in preparation for impending state examinations may be having a negative effect on postural stabilization.

Ultimately, the assessment of FMS and FMS™, as measured in the present cross-sectional study, provides a more robust evidence base for the potential development of an adolescent movement-based intervention. Indeed, creating a change in PA behaviour and movement skill proficiency during adolescence requires a multi-faceted approach [59,60], with the necessity of creating developmentally appropriate activities [34,61,62,63,64] that positively impact movement proficiency. The availability of successful evidence-based programmes targeting motor development, particularly in the early childhood and pre-pubescent literature, has paved the way for the implementation of other FMS movement-oriented interventions to address the identified needs within specific populations [65,66,67,68]. Indeed, actual movement skills are one of the few modifiable risk factors for the prevention of poor health outcomes [26], and therefore promoting movement skill proficiency is integral to a holistic view of development [69]. Furthermore, previous research informed data on functional movement, as measured by the FMS™, suggests that structured interventions lead to positive movement-based outcomes [70], although the most effective ways to develop these movement patterns in Irish adolescent youth is yet to be elucidated. Movement interventions must consist of planned movement activities that are developmentally and instructionally appropriate [62]. There does not appear to be a ceiling effect for both FMS and FMS™ from the current data presented, which necessitates the importance of adolescent intervention implementation for movement. 

It is intended that these baseline findings will help inform the design and development of a larger, movement-oriented intervention, at a later stage. Schools and PE are key potential vehicles for the promotion and provision of movement-based opportunities [8,65]. There is considerable data to suggest that the prescription of FMS programmes during PE may significantly enhance movement skill proficiency [67,68,71,72]. Essentially, the PE environment is a key opportunity to intervene because of access to children and adolescents, that is, for the purpose of improving movement skill proficiency in Ireland [68]. Pedagogical factors such as adequate time set aside to practice the skill, optimal equipment-to-student ratios, specific skill instruction, and effective feedback and encouragement should also be standard practice in the teaching and learning process [40]. It is hoped that through increased emphasis in schools and the wider educational environment, children may develop these movement skills and patterns for heightened PA participation through their lives [40].

A potential limitation of this research is the cross-sectional nature of the study, while the convenience sample of adolescents in this baseline study was limited to just two post-primary schools in one Irish city. Primary areas for future research should therefore use a longitudinal design to provide more insight into how and why fundamental and functional movement may regress with age. Age in the current study was classified using the year of enrolment in school, and not biological age, and therefore any potential findings cannot be generalised to other adolescent populations. Further studies, including larger cross-sectional studies and controlled trials, are required to extend the evidence base and determine whether FMS and FMS™ proficiency change over time, or throughout maturation without intervention, or if movement proficiency changes in response to standardized intervention programs [73].

## 5. Conclusions

Whilst further research is warranted, it appears that school year group is significantly associated with the mastery of movement skills and patterns. The results from the current study, particularly the significant decline apparent in certain skills and patterns, suggest that developing a specifically tailored movement-oriented intervention would be a strategic step towards improving the overall low levels of adolescent fundamental and functional movement proficiency.

## Figures and Tables

**Table 1:** Proportion (%) of males and females in each school year group who correctly performed each component of each fundamental movement skill (FMS).

FMS Component	Males			Females		
	Year 1 (*n* = 46)	Year 2 (*n* = 28)	Year 3 (*n* = 34)	Year 1 (*n* = 33)	Year 2 (*n* = 15)	Year 3 (*n* = 25)
**Catch**						
(1) Preparation phase where hands are in front of the body and elbows are flexed.	93.5	96.4	91.4	97.0	100	100
(2) Arms extend while reaching for the ball as it arrives.	91.3	78.6	85.7	93.9	100	100
(3) Ball is caught be hands only.	97.8	85.7	80.0	87.9	100	100
**Run**						
(1) Arms move in opposition to legs, elbows bent.	91.3	89.3	91.7	81.8	86.7	89.3
(2) Brief period where both feet are off the ground.	100	100	100	100	100	100
(3) Narrow foot placement landing on heel or toe.	97.8	100	97.2	97.0	100	100
(4) Non-support leg bent approximately 90 degrees.	82.6	89.3	88.9	87.9	80.0	96.4
**Balance**						
(1) Support leg still, foot flat on the ground.	100	100	97.1	100	100	100
(2) Non-support leg bent, not touching the support leg.	93.5	89.3	94.3	84.8	100	92.3
(3) Head stable, eyes focused forward.	97.8	92.9	68.6	100	100	96.2
(4) Trunk stable and upright.	89.1	92.9	94.3	100	93.3	100
(5) No excessive arm movements.	87.0	85.7	94.3	84.8	93.3	100
**Skip**						
(1) A rhythmical repetition of the step-hop on alternate feet.	89.1	100	94.4	93.9	100	100
(2) Foot of non-support leg carried near surface during the hop phase.	93.5	100	94.4	93.9	100	100
(3) Arms alternately moving in opposition to legs at about the waist level.	60.9	75.0	86.1	69.7	86.7	78.6
**Kick**						
(1) Rapid continuous approach to the ball.	60.9	64.3	55.6	33.3	20.0	40.7
(2) An elongated stride or leap immediately prior to ball contact.	100	96.4	91.7	81.8	66.7	66.7
(3) Non-kicking foot placed even with or slightly in back of the ball.	87.0	92.9	86.1	39.4	46.7	55.6
(4) Kicks ball with instep of preferred foot (shoelaces) or toe.	100	100	94.4	93.9	86.7	96.3
**Dribble**						
(1) Contacts ball with one hand at about the belt level.	63.0	32.1	42.9	36.4	60.0	34.6
(2) Pushes ball with fingertips (not a slap).	95.7	96.4	91.4	84.8	73.3	88.5
(3) Ball contacts surface in front of or to the outside of foot on preferred side.	87.0	71.4	62.9	87.9	86.7	46.2
(4) Maintains control of ball for four consecutive bounces without having to move the feet to retrieve it.	82.6	92.9	88.6	97.0	86.7	88.5
**Strike**						
(1) Dominant hand grips bat above non-dominant hand.	45.7	57.1	69.4	60.6	60.0	72.4
(2) Non-preferred side of body faces the imaginary tosser with feet parallel.	95.7	96.4	88.9	57.6	80.0	82.8
(3) Hip and shoulder rotation during swing.	100	100	100	87.9	60.0	86.2
(4) Transfers body weight to front foot.	73.9	71.4	86.1	60.6	40.0	51.7
(5) Bat contacts ball.	80.4	85.7	88.9	69.7	86.7	72.4
**Vertical Jump**						
(1) Eyes focused forward or upward throughout the jump.	63.0	60.7	67.6	75.8	53.3	76.9
(2) Crouch with knees bent and arms behind the body.	71.7	71.4	73.5	45.5	53.3	57.7
(3) Forceful forward and upward swing of the arms.	34.8	10.7	47.1	42.4	46.7	38.5
(4) Legs straighten in air.	95.7	92.9	97.1	87.9	100	96.2
(5) Land on balls of feet and bend knees to absorb landing.	87.0	92.9	100	97.0	100	100
(6) Controlled landing with ≤1 step any direction.	87.0	100	100	100	100	100
**Throw**						
(1) Wind-up is initiated with downward movement of hand/arm.	97.8	100	100	97.0	93.3	100
(2) Rotates hip and shoulder to a point where the non-throwing side faces the wall.	50.0	39.3	8.6	9.1	6.7	0
(3) Weight is transferred by stepping with the foot opposite the throwing hand.	71.7	71.4	37.1	51.5	26.7	17.2
(4) Follow-through beyond ball release diagonally across the body towards the non-preferred side.	84.8	92.9	57.1	51.5	20.0	34.5
**Horizontal Jump**						
(1) Preparatory movement includes flexion of both knees with arms extended behind body.	87.0	85.7	88.6	69.7	73.3	76.9
(2) Arms extend forcefully forward and upward reaching full extension above the head.	28.3	25.0	14.3	9.1	20.0	0
(3) Take off and land on both feet simultaneously.	84.8	89.3	97.1	87.9	93.3	80.8
(4) Arms thrust downward during landing.	89.1	92.9	85.7	69.7	66.7	61.5

**Table 2:** Proportion (%) of participants in each school year group who correctly performed each component of the Functional Movement Screen (FMS™).

FMS™ Component	Year 1 (*n* = 68)		Year 2 (*n* = 41)		Year 3 (*n* = 43)	
**Active Straight Leg Raise**	**L**	**R**	**L**	**R**	**L**	**R**
(1) Knee on floor remains in contact with (i.e., touching) the board.	62.7	53.3	61.4	59.1	66.1	69.4
(2) Leg on floor does not externally rotate at the hip.	85.3	89.3	86.4	95.3	91.9	93.5
**Deep Squat**	**-**		**-**		**-**	
(1) Dowel maximally pressed overhead and aligned over feet. Note lumbar flexion.	77.9		81.4		76.7	
(2) Toes point forward.	75.3		86		88.3	
(3) Knees aligned over feet and knees do not go passed the toe line.	72.7		81.4		78.3	
(4) Thighs break parallel with the floor on descent (i.e., femur below horizontal).	77.9		72.1		73.3	
**Hurdle Step**	**L**	**R**	**L**	**R**	**L**	**R**
(1) Hips, knees and ankles aligned.	53.2	53.2	40.9	29.5	46	57.1
(2) Maintains a stable torso with minimal to no movement in lumbar (i.e., lower) spine.	79.7	84.8	86.4	93.2	92.1	93.7
(3) Dowel and hurdle remain parallel.	84.8	89.9	97.7	97.7	96.8	98.4
(4) Foot and/or heel touches the floor while standing leg remains in extended position.	96.2	97.5	97.7	100	100	100
(5) No contact between foot and hurdle.	98.7	100	100	100	100	100
**In-Line Lunge**	**L**	**R**	**L**	**R**	**L**	**R**
(1) Dowel remains in contact with head, thoracic spine and sacrum.	69.2	67.9	65.9	54.5	31.7	36.2
(2) Dowel remains vertical.	70.5	69.2	77.3	65.9	55.6	55.3
(3) No torso movement (i.e., balance is maintained).	91	91	86.4	97.7	92.1	93.6
(4) Knee touches board behind heel of front foot.	85.9	85.9	95.5	93.2	63.5	72.3
(5) The front heel remains in contact with the board and the back heel touches board when returning to starting position.	70.5	66.7	68.2	72.7	69.8	76.6
**Rotary Stability**	**L**	**R**	**L**	**R**	**L**	**R**
(1) Ankles dorsiflexed (i.e., toes tucked under).	9.5	14.9	13.6	18.2	19	17.5
(2) Back remains flat (i.e., spine remains parallel to board).	81.1	86.5	84.1	81.8	87.3	82.5
**Shoulder Mobility**	**L**	**R**	**L**	**R**	**L**	**R**
(1) Does not walk hands towards each other (i.e., one single motion).	83.3	89.2	85.4	90.2	100	98.4
(2) Head remains in neutral position.	76.4	77	76.9	79.5	93.7	92.1
**Trunk Stability Push-Up**	**-**		**-**		**-**	
(1) Body lifts as a unit with no lag in lumbar (i.e., lower) spine when performing the push-up (i.e., chest and stomach come off the floor at the same instance).	24.7		31.8		25	
(2) Ankles are dorsiflexed in both the preparatory and performance phases of the movement.	42.9		47.7		45	
